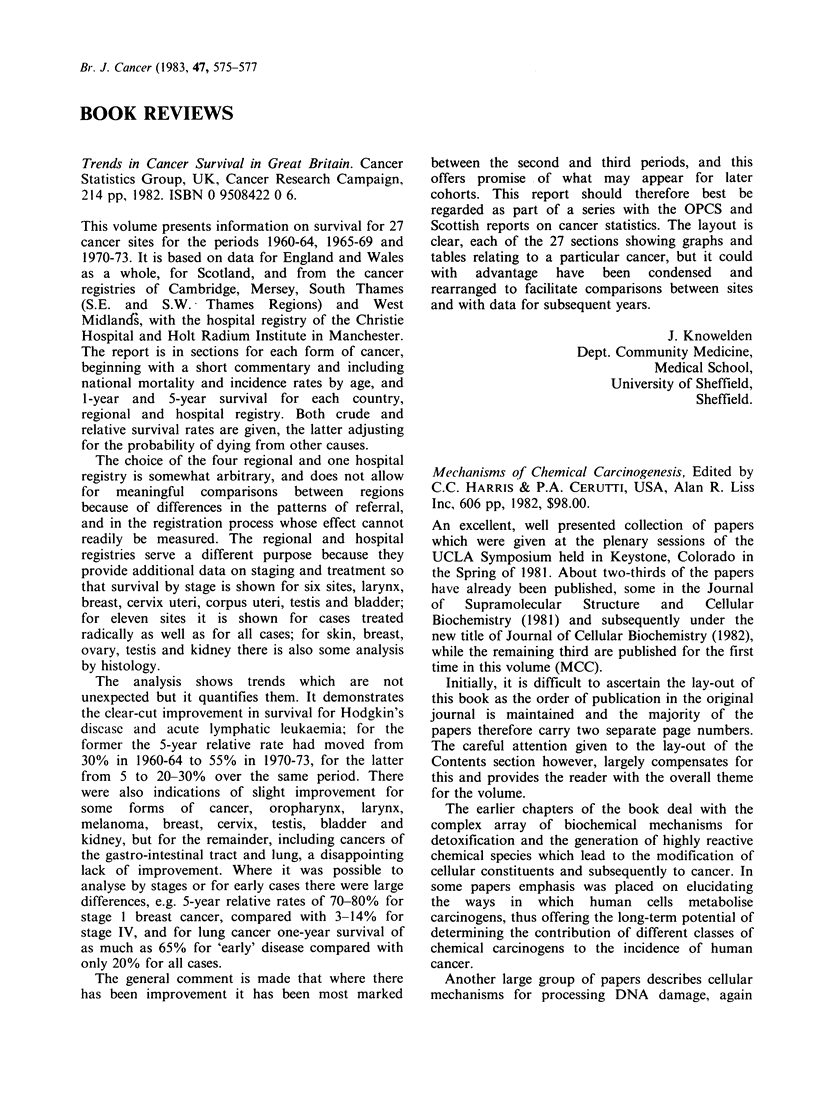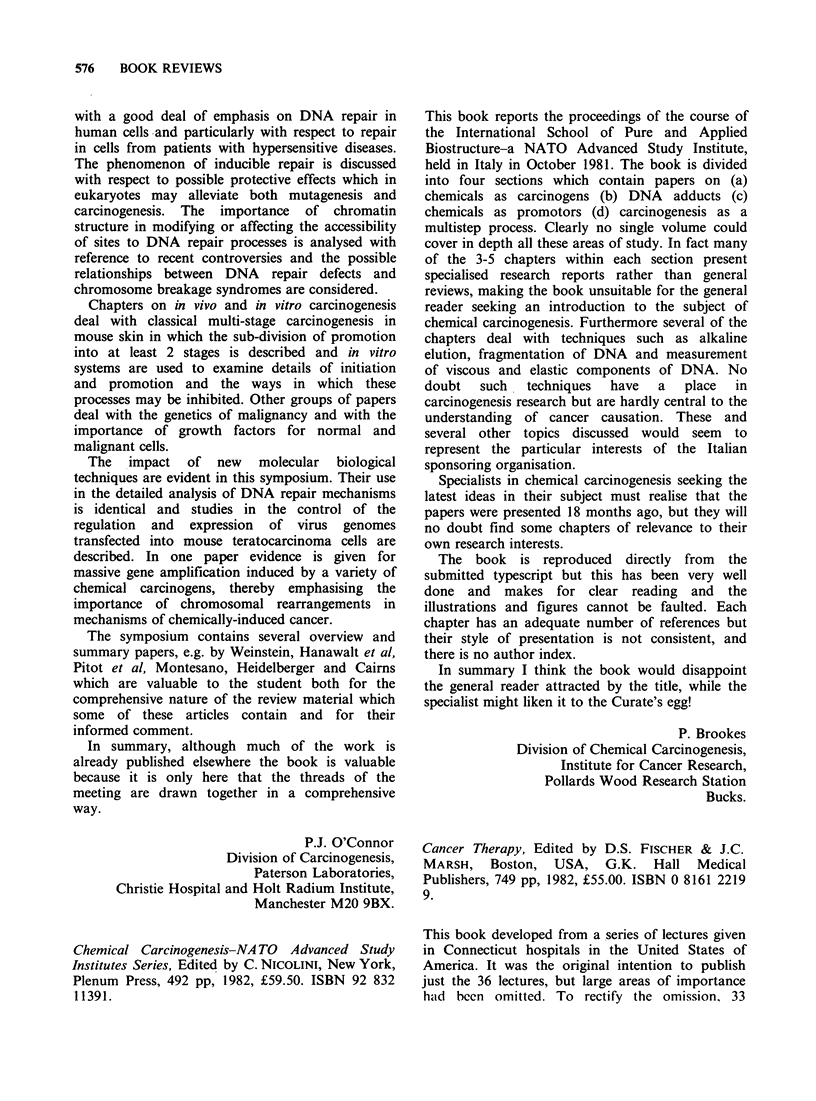# Mechanisms of Chemical Carcinogenesis

**Published:** 1983-04

**Authors:** P.J. O'Connor


*Mechanisms of Chemical Carcinogenesis*, Edited by C.C. Harris & P.A. Cerutti, USA, Alan R. Liss Inc, 606 pp, 1982, $98.00.

An excellent, well presented collection of papers which were given at the plenary sessions of the UCLA Symposium held in Keystone, Colorado in the Spring of 1981. About two-thirds of the papers have already been published, some in the Journal of Supramolecular Structure and Cellular Biochemistry (1981) and subsequently under the new title of Journal of Cellular Biochemistry (1982), while the remaining third are published for the first time in this volume (MCC).

Initially, it is difficult to ascertain the lay-out of this book as the order of publication in the original journal is maintained and the majority of the papers therefore carry two separate page numbers. The careful attention given to the lay-out of the Contents section however, largely compensates for this and provides the reader with the overall theme for the volume.

The earlier chapters of the book deal with the complex array of biochemical mechanisms for detoxification and the generation of highly reactive chemical species which lead to the modification of cellular constituents and subsequently to cancer. In some papers emphasis was placed on elucidating the ways in which human cells metabolise carcinogens, thus offering the long-term potential of determining the contribution of different classes of chemical carcinogens to the incidence of human cancer.

Another large group of papers describes cellular mechanisms for processing DNA damage, again with a good deal of emphasis on DNA repair in human cells and particularly with respect to repair in cells from patients with hypersensitive diseases. The phenomenon of inducible repair is discussed with respect to possible protective effects which in eukaryotes may alleviate both mutagenesis and carcinogenesis. The importance of chromatin structure in modifying or affecting the accessibility of sites to DNA repair processes is analysed with reference to recent controversies and the possible relationships between DNA repair defects and chromosome breakage syndromes are considered.

Chapters on *in vivo* and *in vitro* carcinogenesis deal with classical multi-stage carcinogenesis in mouse skin in which the sub-division of promotion into at least 2 stages is described and *in vitro* systems are used to examine details of initiation and promotion and the ways in which these processes may be inhibited. Other groups of papers deal with the genetics of malignancy and with the importance of growth factors for normal and malignant cells.

The impact of new molecular biological techniques are evident in this symposium. Their use in the detailed analysis of DNA repair mechanisms is identical and studies in the control of the regulation and expression of virus genomes transfected into mouse teratocarcinoma cells are described. In one paper evidence is given for massive gene amplification induced by a variety of chemical carcinogens, thereby emphasising the importance of chromosomal rearrangements in mechanisms of chemically-induced cancer.

The symposium contains several overview and summary papers, e.g. by Weinstein, Hanawalt *et al*, Pitot *et al*, Montesano, Heidelberger and Cairns which are valuable to the student both for the comprehensive nature of the review material which some of these articles contain and for their informed comment.

In summary, although much of the work is already published elsewhere the book is valuable because it is only here that the threads of the meeting are drawn together in a comprehensive way.

P.J. O'Connor
Division of Carcinogenesis,
Paterson Laboratories,
Christie Hospital and Holt Radium Institute,
Manchester M20 9BX.